# Chitosan combined with humic applications during sensitive growth stages to drought improves nutritional status and water relations of sweet potato

**DOI:** 10.1038/s41598-024-55904-x

**Published:** 2024-03-16

**Authors:** Ayman M. S. Elshamly, Rashid Iqbal, Mohamed S. Elshikh, Yasmeen A. Alwasel, Talha Chaudhary

**Affiliations:** 1 Water Studies and Research Complex, National Water Research Center, Cairo, Egypt; 2 Department of Agronomy, Faculty of Agriculture and Environment, The Islamia University of Bahawalpur, Bahawalpur, 63100 Pakistan; 3 Department of Botany and Microbiology, College of Science, King Saud University, 11451 Riyadh, Saudi Arabia; 4 Faculty of Agricultural and Environmental Sciences, Hungarian University of Agriculture and Life Sciences, Godollo, 2100 Hungary

**Keywords:** Climate sciences, Environmental impact, Plant evolution, Plant reproduction, Plant stress responses

## Abstract

The current decline in freshwater resources presents a significant global challenge to crop production, a situation expected to intensify with ongoing climate change. This underscores the need for extensive research to enhance crop yields under drought conditions, a priority for scientists given its vital role in global food security. Our study explores the effects of using humic and chitosan treatments to alleviate drought stress during critical growth phases and their impact on crop yield and water efficiency. We employed four different irrigation strategies: full irrigation, 70% irrigation at the early vine development stage, 70% irrigation during the storage root bulking stage, and 85% irrigation across both stages, complemented by full irrigation in other periods. The plants received either humic treatments through foliar spray or soil application, or chitosan foliar applications, with tap water serving as a control. Our findings highlight that the early vine development stage is particularly vulnerable to drought, with a 42.0% decrease in yield observed under such conditions. In normal growth scenarios, foliar application of humic substances significantly improved growth parameters, resulting in a substantial increase in yield and water efficiency by 66.9% and 68.4%, respectively, compared to the control treatment under full irrigation. For sweet potatoes irrigated with 70% water at the storage root bulking stage, ground application of humic substances outperformed both foliar applications of chitosan and humic in terms of yield results. The highest tuber yield and water efficiency were attained by combining chitosan and humic ground applications, regardless of whether 70% irrigation was used at the storage root bulking stage or 85% irrigation during both the early vine development and storage root bulking stages.

## Introduction

In recent years, while the global population has doubled, this increase has not been matched by a corresponding growth in crop production, leading to frequent food shortages, especially in less developed countries. This disparity highlights the urgent need to identify crops that contribute to food security, particularly those adaptable to diverse ecological environments^1^. Sweet potato (*Ipomoea batatas* L.) stands out as a promising option in this regard due to its rich nutritional profile, including carbohydrates and vitamin A, which are crucial in combating malnutrition^2^. Cultivating sweet potatoes in new, dry-climate regions requires efficient fertilization management, as inadequate nutrient supply can significantly reduce yield^3,4^.

Furthermore, agricultural production is affected by various factors, among which drought is a major challenge, severely impacting global crop production^5^. Climate change has intensified drought conditions, increasing their frequency and severity^6^, with varying effects on crops depending on factors like crop type, drought severity, timing, soil fertility, irrigation management, and other agricultural practices^7–12^.

The growth stages of the sweet potato plant can be divided into four: seedling, fibrous root system establishment, early vine development, and storage root bulking^13^. Research on the drought sensitivity of sweet potatoes is somewhat limited, especially in arid regions. While sweet potatoes are generally tolerant to prolonged drought, they are particularly susceptible during the establishment stage. If drought occurs within the first six weeks after transplanting, it can significantly reduce yield^14–16^. Studies by Zhang et al^17^, and Wang et al^18^, have emphasized the importance of avoiding drought stress during the early growth stages of sweet potatoes. Placide et al^19^, Kivuva et al^20^. found that the plants are sensitive to water shortage at the storage root initiation stage. According to Saraswati^21^, maintaining soil moisture above the wilting point throughout the season is vital for the development of storage roots.

Drought not only affects nutrient availability in the soil by altering their mineralization but also impacts the physiological processes within the plant, influencing nutrient absorption and transfer^22–24^. Jarzyniak & Jasiński^25^, Hussain et al^26^, and Nieves-Cordones et al^27^. attribute this to the reduction in transpiration rates and active transporters, leading to decreased root absorption power and nutrient uptake. In response to drought, plants have developed two primary mechanisms: avoidance and resistance. Avoidance strategies include changes in root and shoot structures, plant growth, and alterations in carbon and nitrogen metabolism. Resistance is often marked by the accumulation of compounds like osmolytes and proteinase inhibitors, which protect plant cells under low water conditions^8^.

Given these challenges, providing appropriate supplements like humic and chitosan can enhance drought resilience, thereby improving crop productivity and contributing to global food security. Humic substances, resulting from the decomposition of plant and animal matter, improve nutrient availability, membrane permeability, enzyme and hormone activity, and water retention, positively affecting plant metabolism and yield^28–36^.

Chitosan applications on crops have been shown to reduce leaf transpiration and improve growth under stress, increasing water absorption and nutrient availability^37–41^. However, the exact mechanisms of chitosan in mitigating adverse conditions are not fully understood^42^.

This study aims to explore the effects of drought on sweet potato growth and yield under various drought conditions, focusing on stages most sensitive to water shortages. It evaluates the effectiveness of applying humic and chitosan, both individually and in combination, to alleviate drought stress. The study also assesses the impact of these treatments on yield, yield quality, and irrigation water efficiency. This research will provide valuable insights into the role of organic fertilizers in sweet potato productivity and nutrition, offering a theoretical basis for optimal irrigation and fertilization strategies during critical drought stages.

## Materials and methods

### Study site

The experiment was carried out during the summer of 2021 and 2022 growing seasons at the experimental farm of water studies and research complex station, National Water Research Center, Toshka, Egypt, which is located at latitude 22°, 24`0.11` N longitude 31°,35`0.43` E and at an altitude of 188 m.

The experimental site is located in southern Egypt and is characterized by a hot and dry summer, a mild winter, and a scarcity of rainfall. Table 1 presents the monthly relative humidity, maximum and minimum temperature, and wind speed in the area during the growing seasons. The main source of irrigation water is groundwater through a well that was dug in the studied area. Table 2 presented ground water chemical properties at the experimental site during (2020–2021) seasons. The physical and chemical properties for the soil are presented in Table 3. All physical and chemical properties of the soil are estimated by following standardized methods of^43,44^. Where, 1.0 g of processed soil sample was weighed and transfer quantitatively into 500 mL Kjeldahl flask. Then (1 g CuSO_4_, 10 g K_2_SO_4_ and 30 mL Con. H_2_SO_4_) was added and stirred well, then nitrogen (N) content was estimated by the Kjeldahl method, as outlined by^43^. Additionally, 1 g of the soil sample was air-dried, sieved (< 2 mm) then added to 10 mL of 0.025 M HCl and 0.03 M NH_4_F and extracted the solution. For 10 min at 2000 rpm, the suspension was centrifuged and filtered. Then by colorimeter methods using a spectrophotometer, the available phosphorous (P) in the filtered extract was then analyzed by measuring absorbance at 660 nm wavelength. On the other hand, 5 g of the soil sample was digested and mineralized with 10 mL mixture of concentrated nitric-perchloric solution for nutrient determination. Then, potassium (K), calcium (Ca), and magnesium (Mg) contents were determined in the previous solution using an atomic absorption spectrophotometer. The sodium (Na) was estimated via flame photometer, while {iron (Fe), manganese (Mn), zinc (Zn), and copper (Cu)} were measured by using atomic absorption spectroscopy. Sulfate anions (SO_4_) were determined by turbidimetric methods using a spectrophotometer at 470-nm wavelength. Bicarbonate anion (HCO_3_) was determined soon after preparing extracts by titration with 0.01 N H_2_SO_4_, while soluble chloride (Cl) was determined by titration with silver nitrate titration. Calcium carbonate (CaCO_3_) was determined by calcimeter method and following the method described by^43^. Electrical conductivity (EC) was measured by extracting the soil sample with water (1:1). To determine soil pH, soil samples were air-dried and sieved through a (1 mm) mesh sieve. Then 10 g of soil sample was taken in a 100 mL beaker then 25 mL of distilled water was added and stirred well. By using digital electrodes (digital ionalyzer/501, Orion research multifunctional pH meter), soil pH was determined. The organic matter of the soil samples was extracted by taking 1g then sieved and added inside a 500 mL Erlenmeyer flask and mixed with (10 mL 1 N potassium dichromate + 20 mL H_2_SO_4_). The mixture was centrifuged gently for about 30 min. Subsequently, 200 mL distilled water + 10 mL H_3_PO_4_) was added, the allow the mixture to cool. Then 15 drops diphenylamine indicator were added and the soil organic matter was estimated via titration with 0.5 M ferrous ammonium sulfate solution.

**Table 1:** Average weather data from the experimental site throughout the period (April to August) during the 2020/2021 growing seasons.

	Temperature (°C)		Relative humidity (%)		Wind speed (MS^−1^)
	Max	Min	Max	Min	
April	39.2 ± 0.20	20.0 ± 0.20	24.2 ± 0.23	3.3 ± 0.05	2.7 ± 0.1
May	40.8 ± 0.19	23.8 ± 0.20	23.5 ± 0.22	3.9 ± 0.02	3.3 ± 0.03
June	41.5 ± 0.19	24.2 ± 0.20	29.9 ± 0.24	4.7 ± 0.01	3.5 ± 0.02
July	43.1 ± 0.19	24.7 ± 0.19	28.8 ± 0.24	5.3 ± 0.05	2.6 ± 0.01
August	43.6 ± 0.20	27.7 ± 0.20	29.5 ± 0.24	6.2 ± 0.02	2.6 ± 0.03

Max maximum temperature, Min minimum temperature, and MS^−1^ m/ second. The meteorological data were obtained from Toshka Agrometeorological Station, Egypt. Values are the mean of replicates ± standard errors.

**Table 2:** Ground water chemical properties at the experimental site, Egypt during the growing seasons of 2020–2021.

Parameter	Unit	Value
pH		6.32 ± 0.31
TDS	mg L^−1^	645.7 ± 0.71
HCO_3_	mg L^−1^	71.0 ± 0.76
Calcium cations (Ca)	mg L^−1^	64.1 ± 0.71
Magnesium cations (Mg)	mg L^−1^	15.8 ± 0.15
Sodium cations (Na)	mg L^−1^	116.5 ± 0.71
Potassium cations (K)	mg L^−1^	4.6 ± 0.09
Chloride anions (Cl)	mg L^−1^	112.3 ± 0.74
Sulfate anions (SO_4_)	mg L^−1^	240.2 ± 0.71

Each value represents the mean of replications ± standard errors. Abbreviations:(TDS) total dissolved solids.

**Table 3:** Some physicochemical properties and water status of soil at the experimental site, Egypt before two successive growing seasons 2020–2021 (mean of 2 years).

Parameter	Unit	Value (cm)	
		0–30	30–60
Mechanical analysis			
Sand	% by weight	90.48 ± 0.71	91.36 ± 0.70
Silt	% by weight	2.56 ± 0.71	2.27 ± 0.71
Clay	% by weight	6.46 ± 0.71	5.57 ± 0.71
Texture	Sand		
Chemical analysis			
pH		6.84 ± 0.72	7.12 ± 0.71
Electrical conductivity (EC) 1:1	ds m^−1^	0.65 ± 0.02	0.39 ± 0.02
TDS	mg L^−1^	416 ± 0.72	250 ± 0.76
CaCO_3_	% by weight	8.30 ± 0.22	7.92 ± 0.24
Calcium cations (Ca)	mg L^−1^	36.07 ± 0.73	24.1 ± 0.74
Magnesium cations (Mg)	mg L^−1^	2.43 ± 0.01	4.86 ± 0.09
Sodium cations (Na)	mg L^−1^	91.9 ± 0.82	45.9 ± 0.75
Potassium cations (K)	mg L^−1^	3.9 ± 0.04	4.0 ± 0.07
Chloride anions (Cl)	mg L^−1^	91.2 ± 0.79	56.7 ± 0.85
Bicarbonate anions (HCO_3_)	mg L^−1^	42.7 ± 1.08	30.5 ± 1.23
Sulfate anions (SO_4_)	mg L^−1^	163.3 ± 0.85	76.8 ± 0.79
Organic matter	% by weight	0.3 ± 0.01	0.2 ± 0.01

EC Electrical conductivity. Carbonate anions (CO_3_) were not detected. Each value represents the mean of replications ± standard errors.

### Experimental design and treatments

The experiment was conducted in a split-plot design with five replications under a drip irrigation system. The experimental units were divided into four main groups representing irrigation regime schemes, i.e.: (A) 100% of the total calculated irrigation at all growth stages which represent normal irrigation conditions, (denoted, normal scheme), (B) 70% of irrigation water at the early vine development stage while applying 100% of the irrigation water at the remaining stages {represent long -term and moderate drought conditions at the early vine development stage, (denoted, Shv 70 scheme), (C) 70% of the irrigation water at the storage root bulking stage while applying 100% of the irrigation water at the remaining growth stages {represent short -term and moderate drought conditions at the storage root bulking stage (denoted, Shs 70 scheme), and (D) 85% of the irrigation water at the early vine development and storage root bulking stages while applying 100% in the remaining growth stages {represent prolonged and mildly drought conditions at the early vine development and storage root bulking stages (denoted, Prv + s 85 scheme). Furthermore, there was a buffer zone between each irrigation unit of 3 m to prevent interactions and each plot was equipped with a manometer valve to control the operating pressure at 1 bar. The plots were also equipped with a flow emitter for discharge with 4.0 L h^–1^ to control the mounts of the targeted irrigation water requirements.

In addition to tap water denoted control which was sprayed by hand sprayer (20 L volume), the plots of each main group were divided into five subgroups representing fertilization applications, which be listed as follows: (a) Chitosan (250 mg L^−1^) was applied as foliar application four times every 15-day interval, initiated after 6 weeks of transplanting, denoted CH; (b) Humic acid (0.5%) was applied as foliar application after 60 and 75 days from transplanting, denoted Hsp, (c) Humic acid as ground drench: 475 (kg ha^−1^) was applied beside the seedling two times after 60 and 75 days from transplanting at two equal doses (273.5 kg ha^−1^ humic for dose), denoted Hgd, (d) Chitosan as a foliar application at a rate of (250 mg L^−1^) in a combination with humic as a foliar application at a rate of (0.5%), denoted CH + Hsp, (e) Chitosan as a foliar application at a rate of (250 mg L^−1^) in a combination with humic as ground drench beside the transplants at a rate of (475 kg ha^−1^), denoted CH + Hgd. The humic was purchased from Egyptian Canadian for humate Co, the product had 65.0% humic substances (involving 13.0% active humic acid and 3% fulvic acid), and 5.0% potassium. While CH: is a high molecular weight that was purchased from Alpha Chemika Co, its solubility reaches about 97% in 1.0% acetic acid under continuous stirring, and the pH was adjusted to 5.6 using 1N NaOH.

It worth to note that CH, Hgd, and Hsp application rates and intervals were implemented according to the manufacturer’s recommendations, and depended on the recommendations of previous study^45–48^

Therefore, based on the aforementioned, the net space of each experimental unit was 45 m^2^ (10.0 m long × 4.5 m width), accordingly, the experimental design involved 100 plots {4 irrigation schemes × 5 fertilizer applications × 5 replicates}.

### The calculations of the irrigation amounts

To calculate daily ETo, the irrigation amounts of the normal scheme were calculated by entering the obtained data from the toshka agrometeorological station, in CROPWAT package, version 8.0, then the crop evapotranspiration of sweet potato (ETc) was calculated according to Allen et al^49^. as the following equation:

$${\text{ETc }} = \, \left( {{\text{ETo }} \times {\text{ Kc}}} \right)$$

where

ETc = Crop evapotranspiration (mm day^−1^).

ETo = Reference evapotranspiration (mm day^−1^).

Kc = Crop coefficient.

Finally, normal scheme irrigation amounts were calculated according to the following equation:

$${\text{IR}} = \frac{{{\text{ETc}} + {\text{Lr}}}}{{{\text{Ei}}}}$$

where

IR = Irrigation requirements (mm).

ETc = Crop evapotranspiration (mm).

Lr = Leaching requirement amounts (%), equaled 10 % since EC of soil solution is low, Lr was neglected.

Ei = Irrigation system efficiency %, the efficiency for drip irrigation system = 85%.

Accordingly, the total of calculated irrigation water amounts under different schemes that applied to the plants during the growing seasons of 2021 and 2022 were: 9346, 8065.5, 8287, and 8175.8 (m^3^ ha^−1^), for normal scheme, Shv 70 scheme, Shs 70 scheme, and (Prv + s 85) scheme, respectively, as demonstrated in Table 4.

**Table 4:** Total irrigation water amounts throughout the different growth stages of sweet potatoes during the growing seasons of 2020/2021.

	Growth stages					
		Seedling & fibrous root establishment	Early vine vegetative	Storage root bulking	Late- season	Total
	Duration (days)	25	70	45	21	
Total irrigation water amounts under different schemes (m^3^ ha ^−1^)	Normal scheme	744.9	4269.0	3531.0	801.3	9346.2
	Shv 70 scheme	744.9	2988.3	3531.0	801.3	8065.5
	Shs 70 scheme	744.9	4269.0	2472.0	801.3	8287.2
	Prv + s 85 scheme	744.9	3628.6	3001.0	801.3	8175.8

Normal scheme (applied 100% of the total calculated irrigation during the all-growth stages, Shv 70 scheme (applied 70% of irrigation water at the early vine development stage while applying 100% of the irrigation water at the remaining stages, represent long -term and moderate drought conditions), Shs 70 scheme (applied 70% of the irrigation water at the storage root bulking stage while applying 100% of the irrigation water at the remaining growth stages, represent short -term and moderate drought conditions), Prv + s 85 scheme (applied 85% of the irrigation water at the early vine development and storage root bulking stages while applying 100% in the remaining growth stages, represent prolonged and mildly drought conditions).

### Crop husbandry

The experiment was prepared as recommended by the Ministry of Agriculture in Egypt for newly reclaimed soil. All treatments were equally fertilized, calcium superphosphate (15.5% P_2_O_5_) was applied to the soil in two portions one during tillage operation at the dose of 360 kg ha^−1^ and the other one at the dose of 240 kg ha^−1^ after 30 days of transplanting. Potassium sulfate (48% K_2_O) at the dose of 288 kg ha^−1^ was applied in three equal portions after 60, 75, and 90 days of transplanting. Nitrogen in the form of ammonium nitrate (33.5% N) at the dose of 360 kg ha^−1^ was applied in three equal portions after 60, 75, and 90 days of transplanting. The sweet potato transplant (*Beauregard cv*) was conducted in the second week of April, during the two study seasons. The source of stem cuttings was the Egyptian company for sweet potatoes, Dakahlia Governorate, Egypt. The Beauregard cultivar is recommended as the high-yielding commercial cultivar. Moreover, this cultivar and the methodologies used in this study were consistent with international, national, and institutional guidelines and legislation. The transplants were approximately 20–25 cm long and were grown on the ridges of the plots, and cultivated using 25 cm spacing between transplants and 70 cm spacing between rows. After 140 days of transplanting the harvesting was done in both seasons.

### Measurements

1-the following data were recorded: A sample of 3 plants from each treatment was randomized 100 days after transplanting to measure the percentage of leaf relative water content (RWC) in sweet potato leaves according to Afzal^50^ using the following formula:

$${\text{RWC}} = \frac{{{\text{FW}} - {\text{DW}}}}{{{\text{TW}} - {\text{DW}}}} \times 100$$

where

FW: Actual weight of sweet potato leaves.

DW: Dry weight of sweet potato leaves.

TW: Turgid weight of sweet potato leaves.

Additionally, the leaves from the top of the sweet potato plants were randomly collected in each plot. Then, the proline content was estimated using the leaves of the plant as described by Luo et al^51^. On the other hand, Nitrogen (N), phosphorus (P) and potassium (K) (%) was determined in the dried leaves according to^52–54^, where the dried leaves were weighed and ground into a fine powder, then N contents were estimated by the Kjeldahl method, P contents were estimated by colorimeter methods using a spectrophotometer at 410-nm wavelength, and K contents were estimated using a flame photometer.

2-At harvest, the following data were recorded:

A-Yield and its components:

Vine fresh weight (kg m ^−2^), tuber roots weight (kg plant ^−1^), and total tuberous roots yield (kg ha^−1^): sweet potato tuberous roots weight taken on plot bases (kg).

B-Tuberous root quality:

To determine tuberous root quality five uniformly sized tuber roots from each treatment were cleaned, cut, dried, and ground. Then weighted for analysis:

- N, P, and K (%) in sweet potato tubers were determined by using the same methods as described above in leaves.
- Total carbohydrates: It was estimated by El-Katony et al^55^. methods. Briefly, 0.5 g of the powdered tissue samples were extracted overnight with 5 mL of 80% ethanol, then for 10 min the extract was centrifuged, replicated with fresh ethanol, and mixed then dried under the vacuum. After that the residue was redissolved in 1 ml of distilled water. The soluble carbohydrate was measured using the glucose calibration curve.
- Protein: The protein of each treatment was recorded by multiplying the total nitrogen by the factor of 6.25 according to Chang and Zhang^56^.
- Soluble sugar: was determined according to Adu-Kwarteng^57^ by adding 10 mL of 80% aqueous ethanol to the tubes and incubating at 80–85 °C for 10 min to extract the soluble sugars, with intermittent mixing on a vortex stirrer. Then for 10 min at 1000 × g (3000 rpm), the tubes were centrifuged. Later, the supernatants were carefully poured off into 50-ml beakers; and re-suspended in another 10 mL of 80% ethanol with repeated this process. Then the supernatants were pooled to obtain the total extracts of soluble sugars, and the remain standard steps were followed.
- Carotene content: was estimated by using a spectrophotometer according to the method described by Qiang et al^58^.

### Irrigation water use efficiency (IWUE)

Mathematically **(**IWUE**)** according to Elshamly^59^, can be estimated by

$${\text{IWUE}} = { }\left( {\frac{{{\text{GY}}}}{{{\text{IW}}}}} \right)$$

where

IWUE = Irrigation water use efficiency (kg m^−3^)

GY = Yield (kg ha^−1^) and

IW = Total calculated irrigation water (m^3^ ha^−1^).

### Statistical analysis

To determine the statistical differences between the treatments CoStat software version 6.303 was used Costat^60^, The means were separated through a revised least significant difference (LSD) test at the 0.05 level as per Casella^61^. At *p* ≤ 0.05, bars that have different letters are statistically significant. Moreover, different lowercase letters on error bars show statistically significant differences either for irrigation schemes (main plot treatments) or examine applications of chitosan and humic acid treatments (subplot treatments) or both.

### Ethics approval and consent to participate

This manuscript is an original paper and has not been published in other journals. The author agreed to keep the copyright rule.

## Results

### The individual and interaction effects of various irrigation schemes and (CH, Hsp, and Hgd) applications on:

#### N concentration in leaves and tuberous

**Figure 1:**
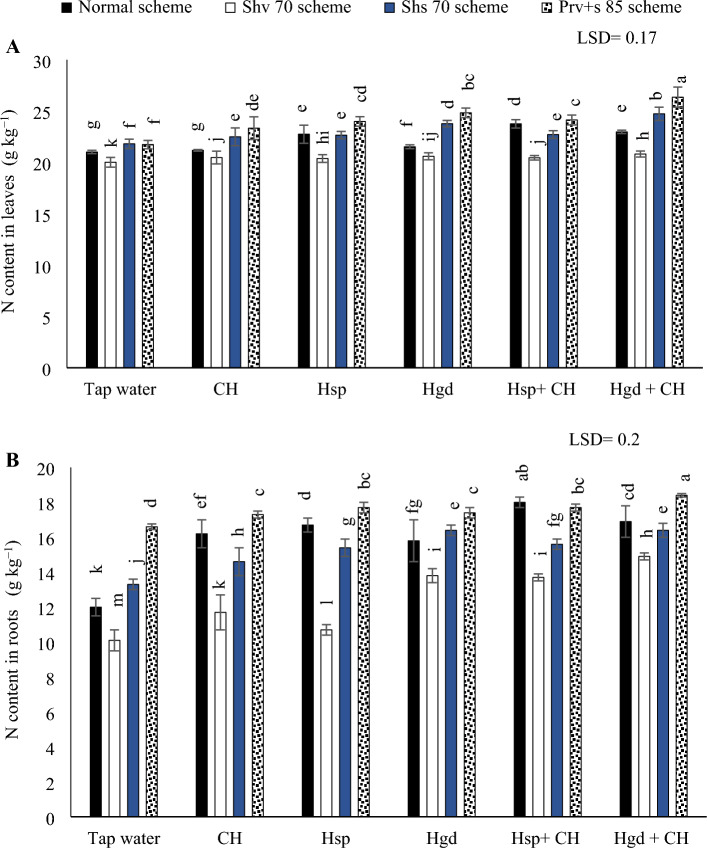
The interactive impacts for the individual or combined application of humic & chitosan application under normal and drought conditions on N (nitrogen content in leaves) (**A)** and N (nitrogen content in tubers) (**B)**. The illustrated values in the figures are the average of the summer of 2021 and 2022 growing seasons. Vertical bars represent ± standard error (SE) of the means. Bars with different letters are statistically significant at *p* ≤ 0.05. Abbreviations: Tap water (spray water- control); CH (spray chitosan); Hsp (foliar applied of humic); Hgd (ground drench of humic); Hsp + CH (foliar applied of humic + chitosan); Hgd + CH (ground drench of humic + foliar applied of chitosan). Normal scheme (applied 100% of the total calculated irrigation during the all-growth stages, Shv 70 scheme (applied 70% of irrigation water at the early vine development stage while applying 100% of the irrigation water at the remaining stages, represent long -term and moderate drought conditions), Shs 70 scheme (applied 70% of the irrigation water at the storage root bulking stage while applying 100% of the irrigation water at the remaining growth stages, represent short -term and moderate drought conditions), Prv + s 85 scheme (applied 85% of the irrigation water at the early vine development and storage root bulking stages while applying 100% in the remaining growth stages, represent prolonged and mildly drought conditions).

The analysis of variance results (ANOVA) for the sole and interaction impacts on the investigated parameters demonstrated that there were significant differences in available N concentration in leaves as a consequence of the individual and interactions impacts. Table 5 showed the individual effects of adopting different irrigation water schemes and different applications of (CH and H) on the average N concentrations during both growing seasons, while (Fig. 1) showed the interaction impacts. In (Fig. 1A), by comparing the various irrigation water schemes in the tap water treatment, choosing Shv70 irrigation water scheme, results in a significant reduction in N contents in the sweet potato leaves compared to the other irrigation schemes. Additionally, it was found that by comparing the impacts of examined applications on N concentration in sweet potato leaves, the solitary applications of Hsp attained higher N concentration by adopting normal irrigation scheme. While there were insignificant variations among the solitary applications of CH and control under the same irrigation scheme. Conversely, it was positive a significant variation could be achieved by adopting Hgd applications. Likewise, under the other irrigation schemes, it was found that the solitary applications of (Hsp and Hgd under Shv70 scheme or Prv + s 85 scheme) and (Hgd under Shs70 scheme) were significantly attained the better N concentrations. Concerning the interaction, the obtained results indicated that there were positive significant impacts by applying Hsp + CH applications under the normal irrigation scheme and applying Hgd + CH applications under the various stress irrigation schemes (Shv70, Shs70, and Prv + s 85 schemes). The findings showed that the combined applications of (Hgd + CH) under Prv + s 85 scheme attained the highest N concentrations in sweet potato leaves**.** While the lowest N contents were observed by applying tap water applications under Shv70 scheme.

**Table 5:** The individual effects of adopting different irrigation water schemes and different applications of (chitosan and humic) on the average nutrient uptake values in the sweet potatoes at the growing seasons of 2020/2021.

Studied factors	N in leaves (g kg^−1^)	N in roots (g kg^−1^)	P in leaves (g kg^−1^)	P in roots (g kg^−1^)	K in leaves (g kg^−1^)	K in roots (g kg^−1^)
Irrigation schemes						
Normal scheme	22.21c	15.998b	3.134c	1.702c	25.913c	16.39c
Shv 70 scheme	20.46d	12.51d	2.974d	1.495d	23.443d	14.46d
Shs 70 scheme	23.0b	15.30c	3.493b	2.419b	27.673a	21.14a
Prv + s 85 scheme	24.07a	17.51a	3.650a	2.609a	26.63b	18.973b
LSD 5%	0.13	0.20	0.0724	0.023	0.1429	0.0975
Applied application						
Tap water (control)	21.15f.	13.07d	3.088d	1.534f.	2.2435f.	15.755f.
CH	21.87e	14.925c	3.167c	1.831e	2.4655e	16.80e
Hsp	22.15d	14.94c	3.219c	2.01d	2.543d	17.415d
Hgd	22.99b	16.015b	3.406b	2.179c	2.6545c	18.36c
CH + Hsp	22.57c	16.125b	3.459b	2.251b	2.7605b	18.675b
CH + Hgd	23.92a	16.93a	3.539a	2.534a	2.882a	19.44a
LSD 5%	0.16	0.162	0.0728	0.026	0.01887	0.1785

Abbreviations: Shv 70 scheme (applied 70% of irrigation water at the early vine development stage while applying 100% of the irrigation water at the remaining stages, represent long -term and moderate drought conditions), Shs 70 scheme (applied 70% of the irrigation water at the storage root bulking stage while applying 100% of the irrigation water at the remaining growth stages, represent short -term and moderate drought conditions), Prv + s 85 scheme (applied 85% of the irrigation water at the early vine development and storage root bulking stages while applying 100% in the remaining growth stages, represent prolonged and mildly drought conditions), N (Nitrogen), P (phosphorus) and K (potassium).

Based on the results in (Fig. 1B), executing Shv70 scheme under different examined applications attained the greater decreases in N concentration in the sweet potato tubers. By comparing the solitary applications of (CH, Hsp, and Hgd), applying solitary applications of Hgd led to attain the best increases in N concentration under (Shv70 and Shs70 schemes), relative to tap water treatment. Likewise, relative to tap water treatment, applying any solitary applications of (CH, Hsp, and Hgd) led to attain the best increases in N concentration under Prv + s 85 scheme. While under the normal scheme, N content attained the best increases by adding the solitary applications of Hsp applications. On the other hand, regarding the interaction impacts of the examined applications, the results indicated that there were positive significant impacts by applying Hsp + CH applications under the normal irrigation scheme and applying Hgd + CH applications under the remaining stress irrigation schemes. Generally, adopting the (Prv + s 85 scheme) and applying combined applications of (Hgd + CH) achieved the maximum increase of N concentration in sweet potato tubers, although that significantly equaled the adoption of the normal irrigation scheme x combined application of Hsp + CH. Similar to N concentrations in sweet potato leaves, the findings indicated that the lowest N contents in tubers were observed by applying tap water applications under the Shv70 scheme.

#### P concentration in leaves and tuberous

**Figure 2:**
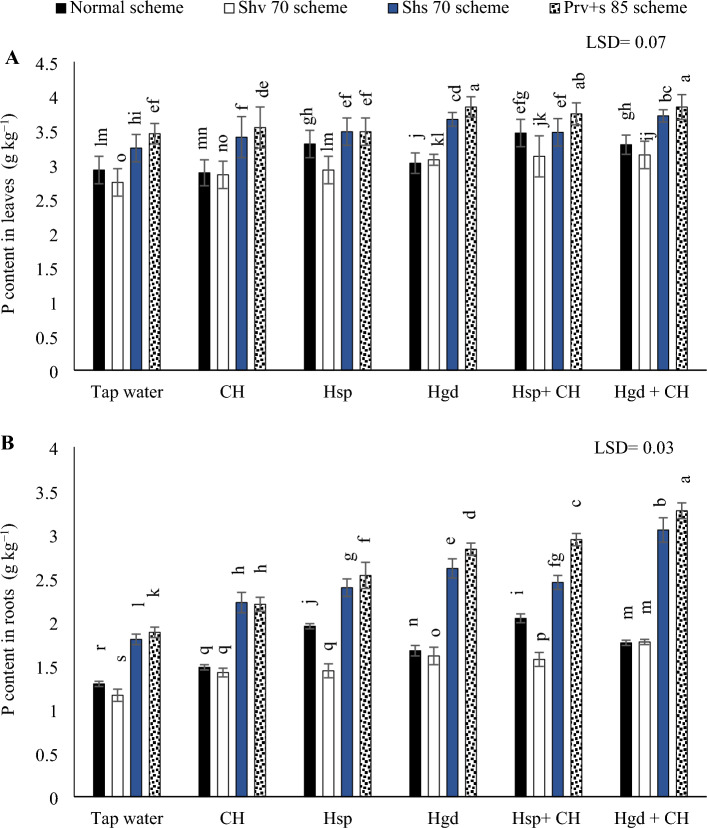
The interactive impacts for the individual or combined application of humic & chitosan application under normal and drought conditions on P (phosphorus content in leaves) (**A)** and P (phosphorus content in tubers) (**B)**. The illustrated values in the figures are the average of the summer of 2021 and 2022 growing seasons. Vertical bars represent ± standard error (SE) of the means. Bars with different letters are statistically significant at *p* ≤ 0.05. Abbreviations: Tap water (spray water- control); CH (foliar applied of chitosan); Hsp (foliar applied of humic); Hgd (ground drench of humic); Hsp + CH (foliar applied of humic + chitosan); Hgd + CH (ground drench of humic + foliar applied of chitosan). Normal scheme (applied 100% of the total calculated irrigation during the all-growth stages, Shv 70 scheme (applied 70% of irrigation water at the early vine development stage while applying 100% of the irrigation water at the remaining stages, represent long -term and moderate drought conditions), Shs 70 scheme (applied 70% of the irrigation water at the storage root bulking stage while applying 100% of the irrigation water at the remaining growth stages, represent short -term and moderate drought conditions), Prv + s 85 scheme (applied 85% of the irrigation water at the early vine development and storage root bulking stages while applying 100% in the remaining growth stages, represent prolonged and mildly drought conditions).

The analysis of variance results (ANOVA) for the sole and interaction impacts on the investigated parameters demonstrated that there were significant differences in P concentration due to irrigation schemes and examined applications. Table 5 showed the individual effects of adopting different irrigation water schemes and different applications of (CH and H) on the average P concentrations during both growing seasons, while (Fig. 2) showed the interaction impacts. By comparing the different irrigation water schemes in the tap water treatment (Fig. 2A), choosing Shs70 or (Prv + s 85) irrigation water schemes, results in a significant enhancement for *P* contents in the sweet potato leaves compared to the normal irrigation schemes. Whereas *P* concentration was increased compared to the normal irrigation scheme by 11.0 and 18.2%, respectively, in the tap water treatment. Conversely, there was a significant reduction by 6.2% could be obtained by adopting Shv70 irrigation scheme relative to the normal irrigation scheme under the same treatment. By comparing the solitary impacts of examined applications on *P* contents in sweet potato leaves, it was found that the solitary applications of Hsp attained higher *P* concentration by adopting normal irrigation scheme. While under the Shv70 scheme, there were positive significant variations by applying both solitary applications of (Hsp and Hgd). Finally, it was positive a significant variation could be attained by adopting Hgd applications under (the Shs70 scheme or Prv + s 85 scheme). Concerning the interaction, the obtained data showed that there was an equaled positive significant impact by applying both combined applications of (Hsp + CH) and (Hgd + CH) under the normal scheme, Shv70, and Prv + s 85 schemes. Conversely, the best significant improvements (15.4%) could be obtained by applying combined applications of (Hgd + CH) and adopting the Shs70 irrigation scheme relative to the normal scheme under tape water treatment. Overall, the findings showed that under the Prv + s 85 scheme, the highest *P* concentrations in sweet potato leaves could be attained by applying the solitary applications of Hgd. Although that significantly equaled by adopting the same irrigation scheme x applying both combined applications of (Hsp + CH) and (Hgd + CH). While the lowest P contents were observed by applying the both of tap water and CH applications under Shv70 scheme.

According to the findings in (Fig. 2B), by comparing the various irrigation water schemes in the tap water treatment, choosing Shv70 irrigation water scheme, results in a significant reduction in *P* concentration in the sweet potato tubers compared to the other irrigation schemes. On the other hand, by comparing the solitary applications, applying solitary applications of Hgd led to attain the best increases in *P* concentration under (Shv70, Shs70, and Prv + s 85) irrigation schemes, relative to tap water treatment. Conversely, applying solitary applications of Hsp led to attain the best increases in *P* concentration in the sweet potato tubers under the normal irrigation scheme. Also, it was observed that under CH treatment, there were insignificant variations among the plants by executing Shv70 scheme compared to the normal scheme. Concerning the interaction impacts, the obtained results indicated that there were positive significant impacts by applying Hsp + CH applications under the normal irrigation scheme and applying Hgd + CH applications under the various stress irrigation schemes (Shv70, Shs70, and Prv + s 85 schemes). Additionally, in contrast to (Hsp + CH), it was observed that under (Hgd + CH) treatment there were insignificant impacts by executing Shv70 scheme compared to the normal scheme. The findings showed that irrigated sweet potato plants with (Prv + s 85) irrigation scheme and applying combined applications of (Hgd + CH), improved the P concentration and achieved the maximum increase for P concentration in the sweet potato tubers. While the lowest P concentrations were observed by applying tap water applications under Shv70 scheme.

#### K concentration in leaves and tuberous

**Figure 3:**
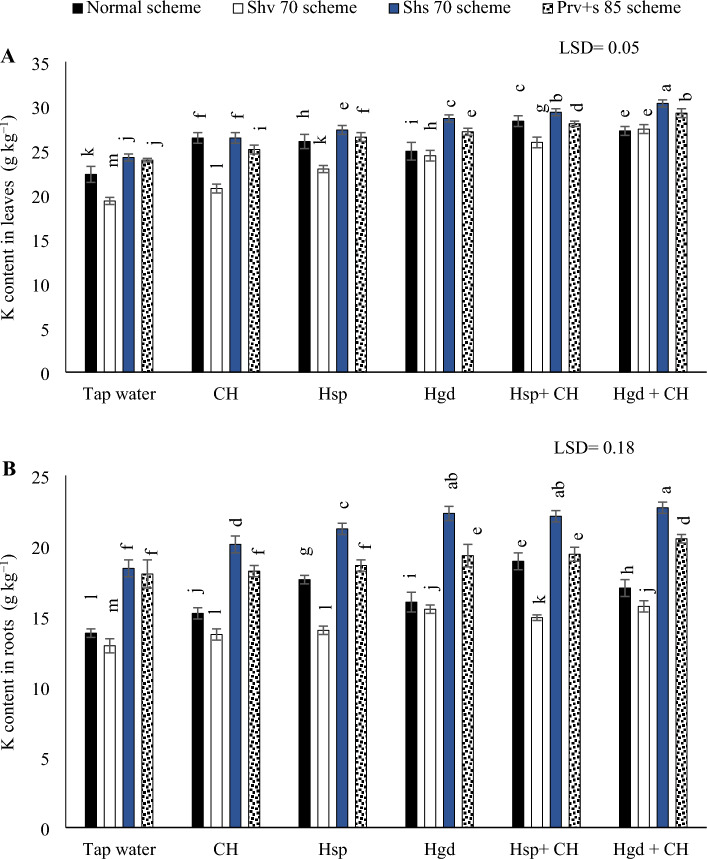
The interactive impacts for the individual or combined application of humic & chitosan under normal and drought schemes on K (potassium content in leaves) (**A)** and K (potassium content in tubers) (**B)**. The illustrated values in the figures are the average of the summer of 2021 and 2022 growing seasons. Vertical bars represent ± standard error (SE) of the means. Bars with different letters are statistically significant at *p* ≤ 0.05. Abbreviations: Tap water (spray water- control); CH (spray chitosan); Hsp (foliar applied of humic); Hgd (ground drench of humic); Hsp + CH (foliar applied of humic + chitosan); Hgd + CH (ground drench of humic + foliar applied of chitosan). Normal scheme (applied 100% of the total calculated irrigation during the all-growth stages, Shv 70 scheme (applied 70% of irrigation water at the early vine development stage while applying 100% of the irrigation water at the remaining stages, represent long -term and moderate drought conditions), Shs 70 scheme (applied 70% of the irrigation water at the storage root bulking stage while applying 100% of the irrigation water at the remaining growth stages, represent short -term and moderate drought conditions), Prv + s 85 scheme (applied 85% of the irrigation water at the early vine development and storage root bulking stages while applying 100% in the remaining growth stages, represent prolonged and mildly drought conditions).

The analysis of variance results (ANOVA) for the sole and interaction impacts on the investigated parameters indicated that there were significant differences as a consequence of the individual and interaction impacts. Table 5 showed the individual effects of adopting different irrigation water schemes and different applications of (CH and H) on the average K concentrations during both growing seasons, while (Fig. 3) showed the interaction impacts. According to the results in (Fig. 3A), by comparing the various irrigation water schemes in the tap water treatment, choosing Shv70 irrigation water scheme, results in a significant reduction in K concentration compared to the other irrigation schemes. While, by comparing the solitary applications of (CH, Hsp, and Hgd), applying solitary applications of Hgd led to attain the best increases in K concentration under the (Shv70, Shs70, and Prv + s 85 schemes), relative to tap water treatment. While under the normal irrigation scheme, applying the solitary applications of CH led to attain the best increases in K concentration in leaves compared to tap water application. On the other side, regarding the interaction impacts of the examined applications, the results indicated that applying the combined applications of Hsp + CH under the normal irrigation scheme led to attain the best increases in K concentration compared to (Hgd + CH) and tap water application. While applying the combined applications of Hgd + CH were pronounced under the (Shv70, Shs70, and Prv + s 85 schemes). Overall, the obtained results showed that K concentration in sweet potato leaves reached the peak by applying the combined applications of Hgd + CH and adopting the Shs70 scheme. On the other hand, adopting the Shv70 scheme and applying tap water application attained the minimum value of K concentration.

On the other hand, the findings in (Fig. 3B) demonstrated that by comparing the various irrigation water schemes in the tap water treatment, choosing Shv70 irrigation water scheme, results in a significant reduction in K concentration compared to the other irrigation schemes. Likewise, by comparing the solitary applications of (CH, Hsp, and Hgd), applying solitary applications of Hgd led to attain the best increases in K concentration under (Shv70, Shs70, and Prv + s 85 schemes), relative to tap water treatment. While, relative to tap water treatment, applying the solitary applications of Hsp led to attain the best increases in K concentration under the normal scheme. On the other hand, regarding the interaction impacts, the results showed that there were positive significant impacts by applying Hsp + CH applications under the normal irrigation scheme. Likewise, applying Hgd + CH application under the (Shv70, Shs70, and Prv + s 85 schemes) (Shv70 and Prv + s 85 schemes), was pronounced to attain the best increases in K concentration. While under Shs70 irrigation scheme there was an equaled positive significant impact by applying both combined applications relative to tap water treatment. In general, relative to the normal scheme under tap water treatment adopting the Shs70 scheme and applying the sole application of Hgd, or either the combined applications of (Hsp + CH) and (Hgd + CH) achieved the maximum increase of K content in sweet potato tubers by (61.6, 60.2, and 64.5%), respectively. While the findings indicated that by adopting the Shv70 scheme and applying tap water application achieved the minimum K concentration in sweet potato tubers.

### The individual and interaction effects of various irrigation schemes and (CH, Hsp, and Hgd) applications on

#### Proline concentration

**Figure 4:**
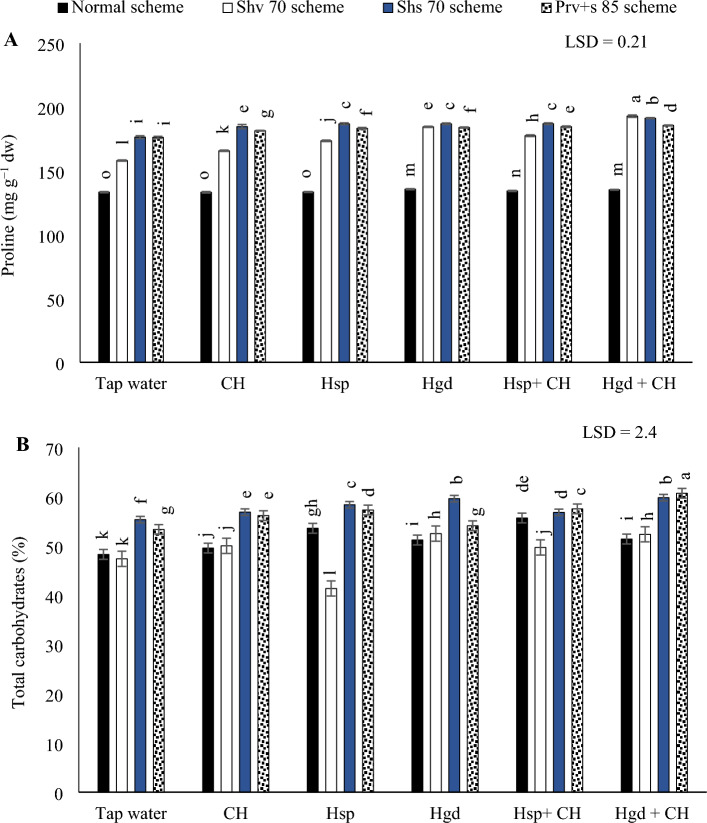
The interactive impacts for the individual or combined application of humic & chitosan under normal and drought schemes on proline (**A)** and total carbohydrates (**B)**. The illustrated values in the figures are the average of the summer of 2021 and 2022 growing seasons. Vertical bars represent ± standard error (SE) of the means. Bars with different letters are statistically significant at *p* ≤ 0.05. Abbreviations: Tap water (spray water- control); CH (spray chitosan); Hsp (foliar applied of humic); Hgd (ground drench of humic); Hgd + CH (ground drench of humic + foliar applied of chitosan). Normal scheme (applied 100% of the total calculated irrigation during the all-growth stages, Shv 70 scheme (applied 70% of irrigation water at the early vine development stage while applying 100% of the irrigation water at the remaining stages, represent long -term and moderate drought conditions), Shs 70 scheme (applied 70% of the irrigation water at the storage root bulking stage while applying 100% of the irrigation water at the remaining growth stages, represent short -term and moderate drought conditions), Prv + s 85 scheme (applied 85% of the irrigation water at the early vine development and storage root bulking stages while applying 100% in the remaining growth stages, represent prolonged and mildly drought conditions).

Table 6 showed the individual effects of adopting different irrigation water schemes and different applications of (CH and H) on the average proline concentrations during both growing seasons, while (Fig. 4A) showed the interaction impacts. Based on the illustrated results in (Fig. 4A), by comparing the different irrigation schemes in the tap water treatment, adopting normal irrigation scheme, resulted in the minimum proline concentration. Conversely, it was shown that the proline concentration could be obtained by adopting the Shs70 or (Prv + s 85) schemes. It was found that by comparing the solitary effects of the examined applications, there were insignificant impact on proline by applying these amendments in a sole application under normal scheme. Conversely, adopting the various stress schemes (Shv70, Shs70, and Prv + s 85 schemes) significantly increased the proline contents in sweet potato leaves. While concerning the interaction, the obtained data showed that there were positive significant impacts by applying Hgd + CH applications under the all the examined irrigation schemes. Overall, the findings showed that the highest proline contents (192.8 mg g^−1^), were obtained by adopting the Shv70 scheme and applying combined applications of (Hgd + CH). however, that significantly equaled the adoption of Shs70 irrigation scheme and applying the same combined application. While the lowest proline values were significantly obtained by applying the full irrigation amounts (normal scheme) and adding (tap water, CH, and Hsp applications).

**Table 6:** The individual effects of adopting different irrigation water schemes and different applications of (chitosan and humic) on the average (proline, carbohydrate, RWC, carotene, soluble sugar, and protein) values in the sweet potatoes at the growing seasons of 2020/2021.

Studied factors	Proline (mg g^−1^ dw)	Total carbohydrate (%)	RWC (%)	Total carotene (mg 100 g^−1^ fw)	Total sugar (%)	Protein (%)
Irrigation schemes						
Normal scheme	134.27d	51.593b	85.145b	5.751b	7.993b	9.903b
Shv 70 scheme	175.75c	48.883c	81.857d	5.507d	6.664d	7.75d
Shs 70 scheme	186.6a	57.796a	86.278a	5.713c	8.661a	9.673c
Prv + s 85 scheme	184.05b	56.513a	84.307c	5.797a	7.822c	11.163a
LSD 5%	0.230	2.304	0.064	0.0217	0.0534	0.180
Applied application						
Tap water (control)	162.15f.	51.084d	82.019f.	5.397f.	7.0325e	8.365d
CH	166.4e	53.1bcd	83.195e	5.609e	7.6125d	9.11c
Hsp	169.7d	52.05 cd	84.529d	5.657d	7.8215c	9.13c
Hgd	171.15c	54.91ab	84.873c	5.7455c	7.9285b	10.09b
CH + Hsp	173.26b	53.84bc	85.689b	5.807b	7.938b	10.145b
CH + Hgd	178.3a	57.134a	86.078a	5.939a	8.379a	10.895a
LSD 5%	0.209	2.589	0.1053	0.0222	0.050	0.160

Abbreviations: Shv 70 scheme (applied 70% of irrigation water at the early vine development stage while applying 100% of the irrigation water at the remaining stages, represent long -term and moderate drought conditions), Shs 70 scheme (applied 70% of the irrigation water at the storage root bulking stage while applying 100% of the irrigation water at the remaining growth stages, represent short -term and moderate drought conditions), Prv + s 85 scheme (applied 85% of the irrigation water at the early vine development and storage root bulking stages while applying 100% in the remaining growth stages, represent prolonged and mildly drought conditions), RWC (leaf relative water content).

#### Carbohydrate content

Table 6 showed the individual effects of adopting different irrigation water schemes and different applications of (CH and H) on the average carbohydrate concentrations during both growing seasons, while (Fig. 4B) showed the interaction impacts. Similarly, by comparing the examined applications under irrigation schemes in tap water treatments, the total carbohydrate content was enhanced when adopting Shs70 or (Prv + s 85) schemes, as can be seen in (Fig. 4B). While there were insignificant variations by executing the Shv70 scheme compared to the normal scheme. By comparing the solitary applications of (CH, Hsp, and Hgd), applying solitary applications of Hgd led to attain the best increases in total carbohydrate content under (Shv70 and Shs70 schemes), relative to tap water treatment. While, applying any solitary applications of Hsp, led to attain the best increases in total carbohydrates content under the normal and Prv + s 85 schemes. On the other hand, regarding the interaction impacts of the examined applications, the data indicated that there were positive significant impacts by applying Hsp + CH applications under the normal irrigation scheme and applying Hgd + CH applications under the remaining stress irrigation schemes (Shv70, Shs70, and Prv + s 85). Generally, adopting the (Prv + s 85 scheme) and applying combined applications of (Hgd + CH) achieved the maximum increase in total carbohydrate concentration. While the findings indicated that the lowest total carbohydrate contents were observed by applying the solitary applications of Hsp under the Shv70 scheme.

#### RWC percentage

**Figure 5:**
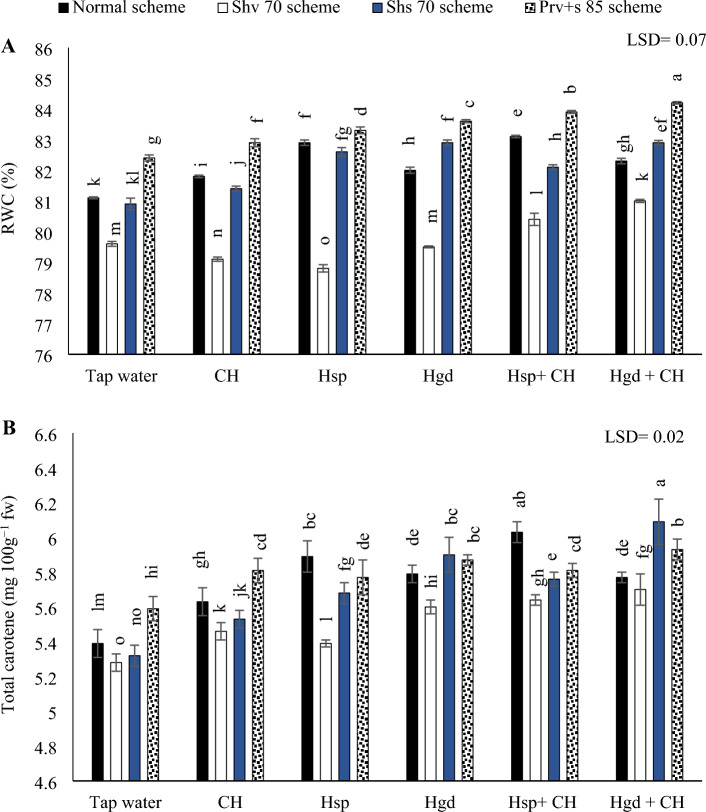
The interactive impacts for the individual or combined application of humic & chitosan under normal and drought schemes on RWC (leaf relative content in sweet potato leaves) (**A)** and total carotene content (**B)**. The illustrated values in the figures are the average of the summer of 2021 and 2022 growing seasons. Vertical bars represent ± standard error (SE) of the means. Bars with different letters are statistically significant at *p* ≤ 0.05. Abbreviations: Tap water (spray water- control); CH (spray chitosan); Hsp (foliar applied of humic); Hgd (ground drench of humic); Hsp + CH (foliar applied of humic + chitosan); Hgd + CH (ground drench of humic + spray chitosan). Normal scheme (applied 100% of the total calculated irrigation during the all-growth stages, Shv 70 scheme (applied 70% of irrigation water at the early vine development stage while applying 100% of the irrigation water at the remaining stages, represent long -term and moderate drought conditions), Shs 70 scheme (applied 70% of the irrigation water at the storage root bulking stage while applying 100% of the irrigation water at the remaining growth stages, represent short -term and moderate drought conditions), Prv + s 85 scheme (applied 85% of the irrigation water at the early vine development and storage root bulking stages while applying 100% in the remaining growth stages, represent prolonged and mildly drought conditions).

Table 6 showed the individual effects of adopting different irrigation water schemes and different applications of (CH and H) on the average RWC concentrations during both growing seasons, while (Fig. 5A) showed the interaction impacts. To maximize RWC percentage in sweet potato leaves, either avoid the Shv70 irrigation scheme or apply combined applications of the examined applications to provide protracted techniques, as can be seen in (Fig. 5A). By comparing the solitary applications, applying solitary applications of Hgd led to attain the best enhancements in RWC percentage under (Shv70 and Prv + s 85) irrigation schemes, relative to tap water treatment. Conversely, applying solitary applications of Hsp led to attain the best increases in RWC percentage in the sweet potato leaves under the normal irrigation scheme. Under the Shs70 scheme, the obtained results showed that both solitary applications of Hsp and Hgd were matched significantly in attaining the best RWC values. Also, it was observed that under CH and Hsp treatments, there were negative significant variations by executing the Shv70 scheme compared to the normal scheme. However, the most negative impacts were observed by applying the solitary applications of Hsp under the Shv70 scheme. While there were insignificant impacts by applying Hgd applications and executing Shv70 scheme compared to the normal scheme. Concerning the interaction impacts, the obtained results indicated that there were positive significant impacts by applying Hsp + CH applications under the normal irrigation scheme and applying Hgd + CH applications under the various stress irrigation schemes (Shv70, Shs70, and Prv + s 85 schemes). The findings showed that by irrigating sweet potato plants with (Prv + s 85) irrigation scheme and applying combined applications of (Hgd + CH), improved the RWC percentage and achieved the maximum increase in the sweet potato leaves. While the lowest RWC percentage was observed by applying Hsp applications under the Shv70 scheme.

#### Carotene content

Table 6 showed the individual effects of adopting different irrigation water schemes and different applications of (CH and H) on the average carotene concentrations during both growing seasons, while (Fig. 5B) showed the interaction impacts. The superiority of (Hgd + CH) applications with normal or Shs70 irrigation schemes remains pronounced leading to an increase in carotene content, as can be seen in (Fig. 5B). By comparing the different irrigation schemes in the tap water treatment, adopting Shv70 and Shs70 schemes were significantly resulted in the minimum carotene content. Conversely, it was shown that the carotene content could be improved by adopting the Prv + s 85 scheme. It was found that by comparing the solitary effects of the examined applications, applying solitary applications of CH and Hgd led to attain the best increases in carotene concentration under the (Prv + s 85) scheme, relative to tap water treatment. While under the normal irrigation scheme, applying the solitary applications of CH led to attain the best increases in carotene concentration compared to tap water application. Under the Shv70 irrigation scheme, applying Hgd was pronounced. While applying the solitary applications of was pronounced by adopting the Shs70 irrigation scheme. While concerning the interaction, the obtained data showed that there were positive significant impacts by applying (Hsp + CH) under the normal irrigation scheme, and (Hgd + CH) applications under the stressful irrigation schemes. Overall, the data showed that the highest carotene contents were obtained by adopting the Shv70 scheme and applying combined applications of (Hgd + CH), although, that significantly matched with adopting the normal irrigation scheme and applying the combined application of (Hsp + CH). While the lowest carotene values were significantly obtained by adopting both irrigation schemes (Shv70 and Shs70) under the tap water treatment.

#### Total sugar content

**Figure 6:**
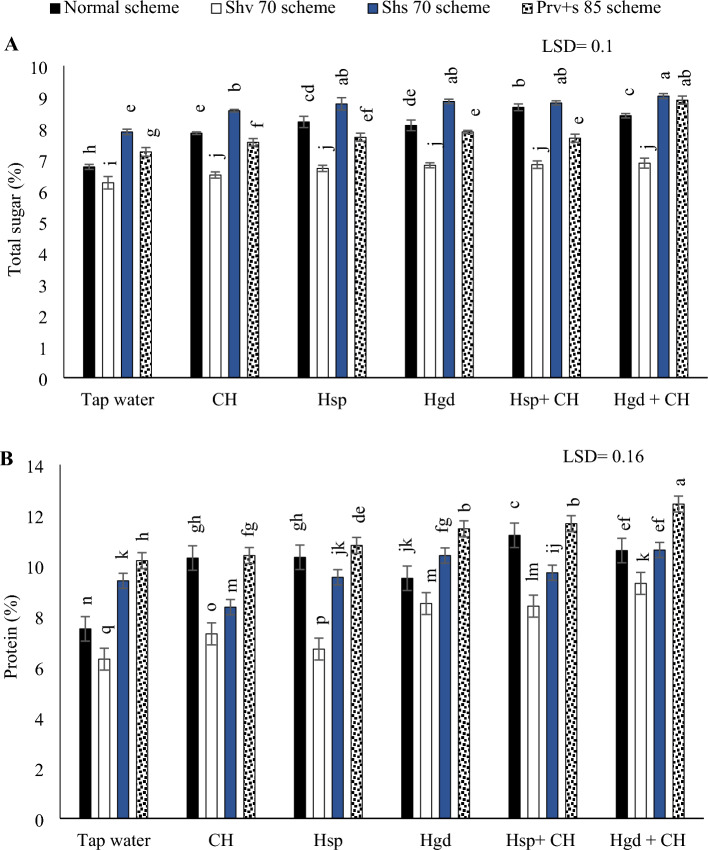
The interactive impacts for the individual or combined application of humic & chitosan under normal and drought schemes on total sugar in tubers (**A)** and protein content in tubers (**B)**. The illustrated values in the figures are the average of the summer of 2021 and 2022 growing seasons. Vertical bars represent ± standard error (SE) of the means. Bars with different letters are statistically significant at *p* ≤ 0.05. Abbreviations: Tap water (spray water- control); CH (spray chitosan); Hsp (foliar applied of humic); Hgd (ground drench of humic); Hsp + CH (foliar applied of humic + chitosan); Hgd + CH (ground drench of humic + spray chitosan). Normal scheme (applied 100% of the total calculated irrigation during the all-growth stages, Shv 70 scheme (applied 70% of irrigation water at the early vine development stage while applying 100% of the irrigation water at the remaining stages, represent long -term and moderate drought conditions), Shs 70 scheme (applied 70% of the irrigation water at the storage root bulking stage while applying 100% of the irrigation water at the remaining growth stages, represent short -term and moderate drought conditions), Prv + s 85 scheme (applied 85% of the irrigation water at the early vine development and storage root bulking stages while applying 100% in the remaining growth stages, represent prolonged and mildly drought conditions).

Table 6 showed the individual effects of adopting different irrigation water schemes and different applications of (CH and H) on the average sugar concentrations during both growing seasons, while (Fig. 6A) showed the interaction impacts. According to the illustrated findings in (Fig. 6A), adopting the Shv70 irrigation scheme, resulted in the minimum sugar content in sweet potato tuberous. While it was found that to increase total sugar percentage, it was better to adopt the Shs70 irrigation scheme under different examined applications. By comparing the solitary applications, applying solitary applications of Hsp and Hgd led to attain the best increases in the total sugar percentage under the (normal, Shs70, and Prv + s 85) schemes, relative to tap water treatment. While, applying any solitary applications of Hsp, led to attain the best increases in the total sugar percentage under the Shv70 scheme. On the other hand, regarding the interaction impacts of the examined applications, the data indicated that there were positive significant impacts by applying Hsp + CH applications under the normal irrigation scheme. While a positive significant impact was matched by applying the both combined applications under the irrigation schemes of (Shv70, Shs70, and Prv + s 85). Generally, adopting the Shs70 scheme and applying either the applications of (Hsp + CH), (Hgd + CH), and Hgd, achieved the maximum increase of the total sugar percentage. Although, that significantly matched with adopting the (Prv + s 85) scheme and applying the combined application of (Hgd + CH). While the findings indicated that the lowest the total sugar values were observed by applying the tap water applications under the Shv70 scheme.

#### Protein content

Table 6 showed the individual effects of adopting different irrigation water schemes and different applications of (CH and H) on the average protein concentrations during both growing seasons, while (Fig. 6B) showed the interaction impacts. Based on the illustrated results in (Fig. 6B), by comparing the different irrigation schemes in the tap water treatment, adopting the Shv70 irrigation scheme, resulted in minimum protein content. Conversely, it was shown that protein content could be obtained by adopting Shs70 or (Prv + s 85) schemes. It was found that by comparing the solitary applications, applying solitary applications of CH and Hsp led to attain the best increases in the protein contents under the normal scheme, relative to tap water treatment. While, applying any solitary applications of Hgd, led to attain the best increases in the protein contents under the (Shv70, Shs70, and Prv + s 85) schemes. While concerning the interaction, the obtained data showed that there were positive significant impacts by applying Hsp + CH applications under the normal scheme. Likewise, there were positive significant impacts by applying Hgd + CH applications under (Shv70, Shs70, and Prv + s 85) schemes. Overall, the findings showed that the highest protein contents (12.44%), were obtained by adopting the (Prv + s 85) scheme and applying combined applications of (Hgd + CH).

### The individual and interaction effects of various irrigation schemes and (CH, Hsp, and Hgd) applications on the yield traits.

**Figure 7:**
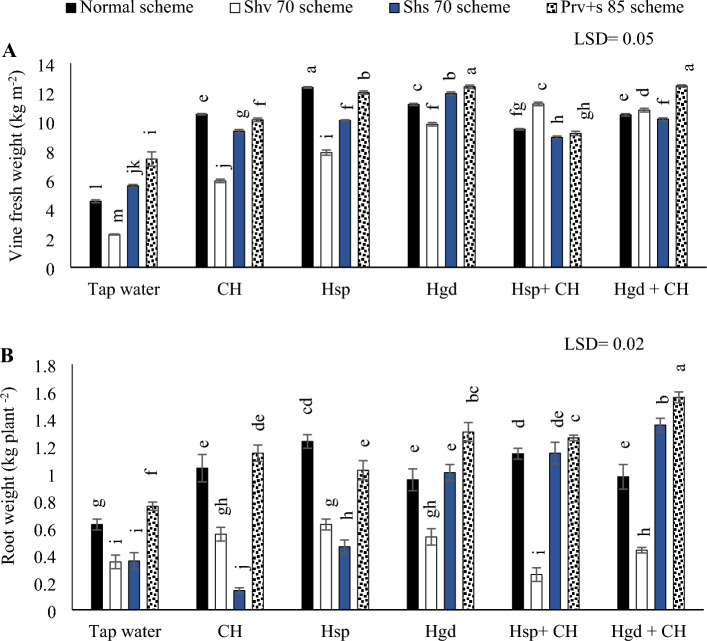
The interactive impacts for the individual or combined application of humic & chitosan under normal and drought schemes on vine fresh weight (**A)** and tuber weight (**B)**. The illustrated values in the figures are the average of the summer of 2021 and 2022 growing seasons. Vertical bars represent ± standard error (SE) of the means. Bars with different letters are statistically significant at *p* ≤ 0.05. Abbreviations: Tap water (spray water- control); CH (spray chitosan); Hsp + CH (foliar applied of humic + chitosan); Hgd + CH (ground drench of humic + spray chitosan). Normal scheme (applied 100% of the total calculated irrigation during the all-growth stages, Shv 70 scheme (applied 70% of irrigation water at the early vine development stage while applying 100% of the irrigation water at the remaining stages, represent long -term and moderate drought conditions), Shs 70 scheme (applied 70% of the irrigation water at the storage root bulking stage while applying 100% of the irrigation water at the remaining growth stages, represent short -term and moderate drought conditions), Prv + s 85 scheme (applied 85% of the irrigation water at the early vine development and storage root bulking stages while applying 100% in the remaining growth stages, represent prolonged and mildly drought conditions).

The individual effects of adopting different irrigation water schemes and different applications of (CH and H) on the average yield traits during both growing seasons are presented in Table 7, while (Fig. 7) showed the interaction impacts.

**Table 7:** The individual effects of adopting different irrigation water schemes and different applications of (chitosan and humic) on the average (yield traits, yield, and IWUE) values in the sweet potatoes at the growing seasons of 2020/2021.

Studied factors	Vine fresh weight (kg m^−2^)	Root weight (kg plant ^−2^)	Root yield (kg ha^−1^)	IWUE (kg m^−3^)
Irrigation schemes				
Normal scheme	9.7394b	1.000b	25,982.1b	2.886b
Shv 70 scheme	8.237d	0.463d	19123c	2.35c
Shs 70 scheme	9.335c	0.7446c	25,980.9b	4.046a
Prv + s 85 scheme	10.316a	1.1750a	31,143.1a	4.146a
LSD 5%	0.0365	0.0177	152.03	0.117
Applied application				
Tap water (control)	4.952f.	0.5841e	15,991.2f.	2.14d
CH	8.9607e	0.7185d	21,661.5e	3.43c
Hsp	9.673d	0.9955a	27,450.3d	3.525bc
Hgd	10.570c	0.9665b	27,834.3c	3.54bc
CH + Hsp	10.964b	0.9101c	28,196.3b	3.595b
CH + Hgd	11.3214a	0.900c	32,210.15a	3.915a
LSD 5%	0.0471	0.0197	147.5	0.129

Abbreviations: Shv 70 scheme (applied 70% of irrigation water at the early vine development stage while applying 100% of the irrigation water at the remaining stages, represent long -term and moderate drought conditions), Shs 70 scheme (applied 70% of the irrigation water at the storage root bulking stage while applying 100% of the irrigation water at the remaining growth stages, represent short -term and moderate drought conditions), Prv + s 85 scheme (applied 85% of the irrigation water at the early vine development and storage root bulking stages while applying 100% in the remaining growth stages, represent prolonged and mildly drought conditions), IWUE (irrigation water use efficiency).

#### Vine fresh weight

Based on the illustrated results in (Fig. 7A), by comparing the solitary applications, applying solitary applications of Hgd led to attain the best enhancements in vine fresh weight under the (normal, Shv70, and Prv + s 85) irrigation schemes, relative to tap water treatment. Conversely, applying solitary applications of Hsp led to attain the best increases in vine fresh weight of the sweet potato leaves under the Shs70 irrigation scheme. Concerning the interaction impacts, the obtained results indicated that there were positive significant impacts by applying Hgd + CH applications under the various irrigation schemes. The findings showed that by irrigating sweet potato plants with (Prv + s 85) irrigation scheme and applying both of the combined applications of (Hsp + CH) and (Hgd + CH), improved the vine fresh weight and achieved the maximum increase. Although, that significantly matched with adopting the normal irrigation scheme and applying the solitary applications of Hsp. While the lowest vine fresh weight was observed by applying tap water applications under Shv70 scheme.

#### Tuber weight

Based on the results in (Fig. 7B), with an exception of the combined applications, executing Shv70 scheme under the examined solitary applications decreased the tuber weight of sweet potatoes. By comparing the solitary applications of (CH, Hsp, and Hgd), applying solitary applications of Hgd led to attain the best increases in tuber weight under (Shs70 and Prv + s 85 schemes), relative to tap water treatment. Likewise, relative to tap water treatment, applying any solitary applications of (CH, Hsp, and Hgd) led to attain the best increases in the tuber weight under the Shs70 irrigation scheme. While under the normal scheme, the tuber weight attained the best value by spraying the solitary applications of Hsp applications. On the other hand, regarding the interaction impacts of the examined applications, the results indicated that there were positive significant impacts by applying Hsp + CH applications under the normal irrigation scheme and applying Hgd + CH applications under the (Shv70, Shs70, and Prv + s 85 schemes) irrigation schemes. Overall, adopting the (Prv + s 85 scheme) and applying combined applications of (Hgd + CH) achieved the maximum increase in the sweet potato tuber weight. While the findings indicated that the lowest tuber weight values were observed by applying CH applications under the Shs70 scheme.

### The individual and interaction effects of various irrigation schemes and (CH, Hsp, and Hgd) applications on

#### Sweet potato yield

**Figure 8:**
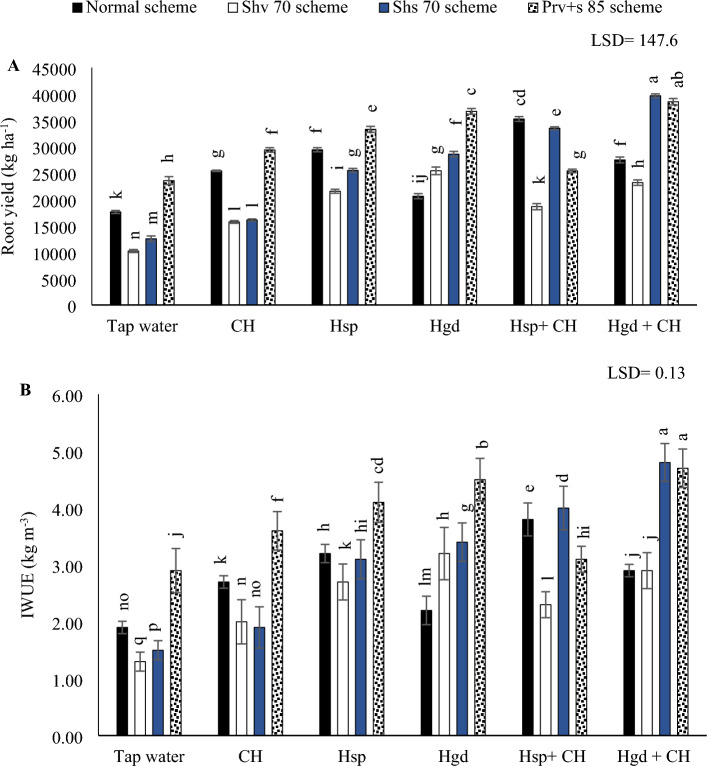
The interactive impacts for the individual or combined application of humic & chitosan under normal and drought schemes on total tuberous yield (**A)** and (IWUE) irrigation water use efficiency (**B)**. The illustrated values in the figures are the average of the summer of 2021 and 2022 growing seasons. Vertical bars represent ± standard error (SE) of the means. Bars with different letters are statistically significant at *p* ≤ 0.05. Abbreviations: Tap water (spray water- control); CH (spray chitosan); Hsp + CH (foliar applied of humic + chitosan); Hgd + CH (ground drench of humic + spray chitosan). Normal scheme (applied 100% of the total calculated irrigation during the all-growth stages, Shv 70 scheme (applied 70% of irrigation water at the early vine development stage while applying 100% of the irrigation water at the remaining stages, represent long -term and moderate drought conditions), Shs 70 scheme (applied 70% of the irrigation water at the storage root bulking stage while applying 100% of the irrigation water at the remaining growth stages, represent short -term and moderate drought conditions), Prv + s 85 scheme (applied 85% of the irrigation water at the early vine development and storage root bulking stages while applying 100% in the remaining growth stages, represent prolonged and mildly drought conditions).

The individual effects of adopting different irrigation water schemes and different applications of (CH and H) on the average sweet potatoes yield during both growing seasons are presented in Table 7, while (Fig. 8A) showed the interaction impacts. The analysis of variance results (ANOVA) for the sole and interaction impacts on the investigated parameters indicated that there were significant differences as a consequence of the individual and interactions impacts on the sweet potato yield. According to the results in (Fig. 8A), to improve sweet potato yield, (A) avoid the adoption of the Shv70 irrigation scheme under the various examined applications except with solitary applications of Hgd; (B) apply combined applications of the (Hgd + CH) and under the both irrigation schemes of Shs70 and (Prv + s 85) to provide protracted techniques; (C) avoid the adoption of the (Hsp + CH) under the Shv70 irrigation scheme. The results indicated that by comparing the various irrigation water schemes in the tap water treatment, choosing Shv70 irrigation water scheme, results in a significant reduction in sweet potato yield compared to the other irrigation schemes. Also, by comparing the solitary applications, applying solitary applications of Hgd led to attain the best enhancements in sweet potato yield under (Shv70, Shs70, and Prv + s 85) irrigation schemes, relative to tap water treatment. Conversely, applying solitary applications of Hsp led to attain the best increases in sweet potato yield under the normal irrigation scheme. On the other hand, regarding the interaction impacts of the examined applications, the results indicated that applying the combined applications of Hsp + CH under the normal irrigation scheme led to attain the best increases in sweet potato yield compared to (Hgd + CH) and tap water application. While applying the combined applications of Hgd + CH under were pronounced under the (Shv70, Shs70, and Prv + s 85 schemes). Overall, the obtained results showed that sweet potato yield reached the peak by applying the combined applications of Hgd + CH and adopting the Shs70 scheme. However, that significantly matched with adopting the (Prv + s 85) irrigation scheme and applying the combined applications of Hgd + CH. On the other hand, adopting the Shv70 scheme and applying tap water application attained the minimum value of sweet potato yield.

#### IWUE

The individual effects of adopting different irrigation water schemes and different applications of (CH and H) on the average IWUE during both growing seasons are presented in Table 7, while (Fig. 8B) showed the interaction impacts. On the other hand, with an exception of the combined application of (Hsp + CH), IWUE was increased by adopting the Prv + s 85 scheme, as can be seen in (Fig. 8B). By comparing the solitary applications of (CH, Hsp, and Hgd), applying solitary applications of Hgd led to attain the best increases in IWUE under the (Shv70, Shs70, and Prv + s 85 schemes), relative to tap water treatment. While under the normal irrigation scheme, applying the solitary applications of Hsp led to attain the best increases in IWUE compared to tap water application. On the other side, regarding the interaction impacts of the examined applications, the data indicated that applying the combined applications of Hsp + CH under the normal irrigation scheme led to attain the best increases in IWUE compared to (Hgd + CH) and tap water applications. While applying the combined applications of Hgd + CH under were pronounced under the (Shv70, Shs70, and Prv + s 85 schemes). The obtained results showed that IWUE of sweet potato reached the peak by applying the combined applications of Hgd + CH and adopting Shs70 or Prv + s 85 schemes. While adopting the Shv70 scheme and applying tap water applications attained the minimum value of IWUE. Overall, the IWUE increased from the lowest recorded value (1.3 kg m^−3^) for the control Shv70 scheme in the tap water treatment to the highest significant values (4.8 and 4.7 kg m^−3^) for (Hgd + CH x Shs70 scheme) and (Hgd + CH x Prv + s 85 scheme), respectively.

## Discussion

While sweet potato is considered a moderately drought-tolerant crop, research into its optimal water and fertilizer management across different growth stages in arid conditions has been largely overlooked. To increase yield and improve IWUE under such challenging environments, it is crucial to implement effective irrigation management strategies. These strategies should be complemented by enhancing the plant's drought tolerance through the application of suitable supplementary treatments, as emphasized by Zhou et al^62^. This approach involves not just regulating water supply but also incorporating additional aids that support the plant's resilience to water scarcity.

### Impacts of CH, Hsp, and Hgd under normal irrigation conditions on tuber yield and IWUE

In this study, we investigated the effects of applying CH, humic acid as a soil drench (Hgd), and humic acid as a foliar spray (Hsp) under normal irrigation conditions. The results revealed that these applications, whether used individually or in combination, significantly influenced growth traits and helped improve IWUE. Specifically, while individual application of Hsp significantly enhanced tuber yield and IWUE, the combination of Hsp and CH proved to be more effective under the same irrigation conditions compared to other treatments. These findings align with Chen et al^63^, who noted that humic acid and chitosan can promote the growth and yield of sweet potatoes.

The study posits that these effects may result from an imbalance in nutrient transport activities, particularly between the plant's aerial parts and roots. The application of Hsp alone seemed to compensate for this imbalance, enhancing nutrient absorption, vine weight, and thereby yielding better results. Man Hong et al^64^.observed a similar phenomenon, noting that Hsp application primarily influenced the aerial parts of potato plants, with a delayed effect on root development. Moreover, the combined application of Hsp and CH under normal irrigation conditions achieved optimal yield and IWUE. This is attributed not only to the benefits of Hsp but also to the nitrogen content in CH. The addition of Hsp, with its acidic components, increased the solubility of CH, amplifying its positive effects. This observation is supported by Li et al^65^, who found that foliar application of humic acid can increase nitrogen contents. Yildirim^66^ also noted the significant impact of foliar humic in enhancing soil nutrient levels by promoting root growth and function, ultimately improving yield, a finding echoed by other studies^39,46,63^. However, it's important to note that when sweet potato plants were irrigated according to their actual water needs (normal scheme), neither solitary nor combined applications achieved the highest yield. This may be due to increased soil water and nutrient contents under these conditions, leading to a shift from productive to vegetative growth (e.g., more leaves, taller vines), as also observed in other studies^62,67^ This indicates that over-ideal conditions, such as over-irrigation or excessive nitrogen application, can lead to unfavorable outcomes like overgrowth of shoots and leaves, immature leaf development, and reduced storage processes in sweet potatoes.

### Impacts of various water stress schemes on sweet potatoes and the role of CH, Hsp, and Hgd under these conditions

This study underscores the importance of avoiding prolonged drought stress in sweet potato cultivation and advocates for a moderate irrigation approach, particularly during the early vine development stage. The results indicate that the Shv70 irrigation scheme (70% of normal water supply during early vine development) leads to significant decreases in growth traits and yield. Consistent with previous findings^17,18^, sweet potatoes, though drought-resistant, are particularly vulnerable during the early vine development stage. The Shv70 scheme appears to push the plants past a critical threshold, adversely affecting their growth. In response to such stress, plants increase proline production, a protective mechanism under stress conditions, aligning with findings in^68^. During the early vine development stage, sweet potatoes prioritize forming a robust root system and then developing strong aerial tissues. However, under stress, these priorities shift, leading to an accelerated life cycle but with an underdeveloped root system, resulting in reduced nutrient absorption and, consequently, lower yields.

In such stressful conditions, an effective fertigation strategy that minimizes drought effects and optimizes yield through the right combination of humic and CH applications is crucial. This study found that applying humic acid as (Hgd) alone, particularly under the Shs70 irrigation scheme (70% water supply during the storage root bulking stage), significantly improved IWUE compared to sole applications of CH and Hsp (humic acid as a foliar spray). This improvement can be attributed to the increased activity of the root system under stress, which becomes the primary driver of plant activities. By applying Hgd to the roots, enhanced nutrient absorption and storage are facilitated, leading to improved crop yield and IWUE, as supported by findings in^69–71^. Additionally, previous studies^64,72^ have shown that humic applications enhance crop resistance to drought stress and improve nutrient uptake and utilization, thereby increasing yield under less favorable water conditions. Humic acid applications have also been noted to improve soil properties in arid and semi-arid areas^73,74^.

Furthermore, the study reveals that the highest tuber yields are achievable through the combined application of Hgd and CH, either under the Shs70 scheme or under a mild irrigation scheme throughout both the early vine development and storage root bulking stages (Prv + s 85). Under the Shs70 scheme, plants enhance (P and K) absorption and proline production, benefiting from an established root system from the previous stage, thereby improving tuber yield. These findings align with research in^75,76^. Moderate irrigation later in the season can slow leaf senescence, extend their functional period, and boost photosynthetic efficiency, as indicated by^62^.

### Feasibility of CH, Hsp, and Hgd in improving sweet potato resistance during the most sensitive stage to drought conditions

In line with the earlier observation about the vulnerability of sweet potatoes to drought during the early vine development stage, the combined application of humic acid as Hgd and CH has shown promise in improving tuber yield. This enhancement can be attributed to the ability of these applications to mitigate drought impacts and create better growth conditions. Specifically, applying CH four times has been found to enhance sweet potato drought resistance. CH contributes to the formation of a transparent layer on the leaves, which reduces the transpiration rate and increases water content, thereby conserving water and enhancing IWUE. Adamuchio-Oliveira et al^77^. support this, indicating that foliar application of CH increases the thickness of the leaf blade, resulting in enhanced water storage within the tissue. Additionally, the amino proton group present in CH boosts photosynthesis rate and nutrient absorption, improving yield quality in terms of carbohydrates and proteins, as corroborated by findings in^38,39,78,79^. Furthermore, supplementing plants with Hgd under the same irrigation conditions amplifies the benefits, enhancing plant resistance to drought and, consequently, improving tuber yield. This is in line with research^46,80^, suggesting that Hgd application not only increases nutrient uptake but also enhances transport efficiency by chelating unavailable nutrients and lowering soil pH, as noted by Mackowiak et al^81^.

These findings highlight the importance of proactively supplying plants with additional substances to fortify them against drought. Given that drought is among the most challenging environmental stresses to predict in terms of duration, occurrence, and severity^82,83^, adopting such strategies is crucial for ensuring crop resilience and maintaining productivity in the face of fluctuating and often harsh environmental conditions.

## Conclusion

This study sheds light on the effects of drought on sweet potato plants, leading to several significant conclusions. Firstly, the early vine development stage of sweet potato plants is highly sensitive to drought conditions. This finding underscores the importance of proactively supplementing these plants with additional substances to bolster their defense against such unfavorable environmental stressors. Secondly, when sweet potatoes are irrigated normally, applying humic acid either alone (as a foliar application) or in combination with chitosan substantially improves both yield and water use efficiency. This observation suggests that even under conditions of adequate water supply, these treatments contribute positively to plant growth and productivity. Thirdly, the study finds that during periods of drought in the storage root bulking stage, using humic acid as a ground drench is more effective than either using chitosan alone or applying humic acid as a foliar spray. This highlights the importance of the application method and timing, particularly in relation to the specific growth stages of the plant, in effectively managing drought stress. Lastly, for achieving the highest yield and water use efficiency, it is crucial to expose sweet potato plants to a mild drought regime during both the early vine development and storage root bulking stages, while applying a combination of chitosan and ground drench humic acid. This approach appears to be the most effective in mitigating the undesirable effects of drought.

## Data Availability

The presented datasets during this study available from the corresponding author on reasonable request.
